# Cardiac magnetic resonance imaging-derived septum swing index detects pulmonary hypertension: A diagnostic study

**DOI:** 10.2478/jtim-2023-0114

**Published:** 2023-12-20

**Authors:** Miao He, Rong Jiang, Fei-Sun, Jing-Xue Cao, Lan Wang, Jing-Yun Shi

**Affiliations:** Department of Radiology, Shanghai East Hospital, School of Medicine, Tongji University, Shanghai 200120, China; Department of Cardio-Pulmonary Circulation, Shanghai Pulmonary Hospital, School of Medicine, Tongji University, Shanghai 200433, China; Jedicare Medical Co. Ltd., Shanghai 201210, China; Department of Radiology, Shanghai Pulmonary Hospital, School of Medicine, Tongji University, Shanghai 200433, China

**Keywords:** cardiac magnetic resonance, septum swing index, pulmonary hypertension, pulmonary hemodynamics

## Abstract

**Background and Objectives:**

Because of pressure differences between the pulmonary artery and aorta, the ventricular septum moves in a swinging motion that is commonly observed on cardiac MR (CMR) cine sequences in patients with pulmonary hypertension (PH). We aimed to assess the use of septum swing index (SSI) derived by CMR for detecting PH.

**Methods:**

We retrospectively identified consecutive patients with suspected PH who underwent right heart catheterization (RHC) and CMR at a PH referral center between July 2019 and December 2020. The diagnostic accuracy of SSI for identifying PH (mean pulmonary artery pressure [mPAP] ≥ 25 mmHg) was assessed by receiver operating characteristic curves, sensitivity, specificity, and positive and negative predictive values.

**Results:**

A total of 105 patients (mean age: 47.8 ± 15.0 years; 68 females) were included in the final analysis. SSI and mPAP were negatively correlated in the total study population and patients with PH, but not in patients without PH. SSI was an independent predictor of PH (adjusted odds ratio: 12.9, 95% confidence interval: 3.6 to 45.5, *P* = 0.003). The area under the curve for SSI was 0.91, with a cut-off value of 0.9673 yielding the best balance of sensitivity (86.4%), specificity (88.2%), positive predictive value (97.4%), negative predictive value (55.6%), and accuracy (86.7%) for detecting PH.

**Conclusions:**

Septum swing index was lower in patients with PH and is a simple, reliable method for detecting PH.

## Introduction

Pulmonary hypertension (PH) is a severe clinical condition characterized by increased pulmonary vascular resistance and right ventricular remodeling, leading to right heart failure.^[1,2]^ Right ventricular function is predictive of the severity of PH, as well as patient exercise capacity and survival, and strong evidence indicates that patients with right ventricular dysfunction experience faster clinical deterioration.^[3, 4, 5, 6]^ However, unlike the left ventricle (LV), the complex chamber geometry and suboptimal endocardial definition of the right ventricle (RV) have limited the application of noninvasive RV function assessment in clinical practice.^[7]^ Additionally, the dynamic and complicated remodeling process of the RV subjected to pressure overload is distinctly different from that of the LV during systemic hypertension.^[5,8–9]^ In patients with PH, the circular shape of the LV on cross-sectional images changes to a “D” shape, and the crescentic RV assumes a more circular shape.^[10]^ Changes in ventricular geometry can be represented by the left ventricular eccentricity index. Recent evidence has shown that this index is associated with myocardial fibrosis and adverse outcomes in patients with PH.^[11, 12, 13]^

Cardiac MR (CMR) imaging is a reliable, reproducible non-invasive method for assessing cardiac structure and function and is considered the standard reference for RV evaluation.^[14, 15, 16, 17]^ Interventricular septum swing is frequently observed on CMR cine sequences of patients with PH. Pathophysiologic changes in PH, with alterations in the normal pressure difference between the pulmonary artery and aorta, lead to changes in the position of the interventricular septum (an elastic tissue between two pressure sources: the RV and LV), as well as deformation of the ventricular chambers. However, no simple quantitative method has been reported describing the link between the degree of ventricular chamber deformation detected by non-invasive MR imaging and pulmonary artery pressure values obtained by invasive, “gold standard” right heart catheterization (RHC).

In this study, we introduce a very simple CMR imaging-based deformation value, which we have called the septum swing index (SSI), and investigate the utility of SSI for assessing RV hemodynamic changes in PH. Specifically, we analyze the correlation between SSI and the mean pulmonary arterial pressure (mPAP) obtained by RHC. We hypothesize that SSI will be an accurate reflection of mPAP alteration and RV remodeling in patients with PH and may play an important role in the long-term monitoring of these patients.

## Materials and methods

The protocol for this retrospective study was approved by our local ethics committee (L21–387), and informed consent was obtained from all patients.

We screened all patients with suspected PH who underwent RHC and CMR at a PH referral center between July 2019 and December 2020. All included patients met the following criteria: (1) World Health Organization (WHO) group 1 or 4 PH based on the 6th World Symposium on PH,^[18]^ including idiopathic, heritable, associated with connective tissue disease, and chronic thromboembolic PH; (2) aged ≥ 18 years; (3) WHO functional class II or III; (4) 6-minute walking distance ≥ 200 m; (5) sinus rhythm < 100 bpm. The exclusion criteria were (1) other WHO PH groups (15) ;(2) contraindications to gadolinium chelates; (3) too weak to tolerate CMR; or (4) declined CMR; (5) arrhythmias, such as atrial fibrillation.

### Hemodynamic measurement

RHC was performed as described previously,^[19]^ The baseline hemodynamic variables evaluated included mPAP, pulmonary artery wedge pressure, cardiac output, cardiac index and pulmonary vascular resistance.

### Cardiac magnetic resonance procedure and image analysis

All CMR images were analyzed by two radiologists who had at least 10 years’ experience with interpreting CMR results and who were unaware of the RHC results (M. H. and J-Y. S.). Endocardial contours of the LV and RV and diastolic and systolic perimeter and area were obtained on CMR images. Stroke volume, end-diastolic volume, end-systolic volume, ejection fraction, and ventricular mass index (VMI) were measured using MASS software package (MEDIS Medical Imaging Systems, Leiden, the Netherlands).^[20, 21, 22]^ Detailed procedures and analyses are presented in the supplementary text. All patients underwent RHC and CMR within 7 days.

### Septum swing index calculation

The senior MRI data expert (F. S.) created the SSI calculation, which consists of three steps:

### Step 1: Calculate the SSI at end-diastole

Select two consecutive slices that show the maximum LV chamber area in the short-axis cine sequence and draw a complete outline of the LV of each slice using any measurement software (*e. g*. Radiant [www.radiantviewer.com]) to obtain the slice area and corresponding slice perimeter. The diastolic SSI (SSI^diastolic^) is calculated using this formula:


$${SSI}_{\text{diastolic }}=\left({SSI}_{\text{slice1 }}+{SSI}_{\text{slice2 }}\right)/2=\frac{\frac{4\pi \times \text{ slice }1\text{ area }}{\text{ slice }1{\text{ perimeter }}^{2}}+\frac{4\pi \times \text{ slice }2\text{ area }}{\text{ slice }2{\text{ perimeter }}^{2}}}{2}$$

### Step 2: Calculate the SSI at end-systole

Repeat the same calculation as step 1 for the end-systolic phase cine sequence to obtain the systolic SSI (SSI^systolic^).

### Step 3: Calculate the final SSI

Based on the RHC-based mPAP formula $\left(\frac{PAP_{\text{systolic }}+2\times PAP_{\text{diastolic }}}{3}\right),$the corresponding final SSI is calculated as follows: SSI = $\frac{SSI_{\text{systolic }}+2\times SSI_{\text{diastolic }}}{3}.$

### SSI numerical approximation

**Figure 1:**
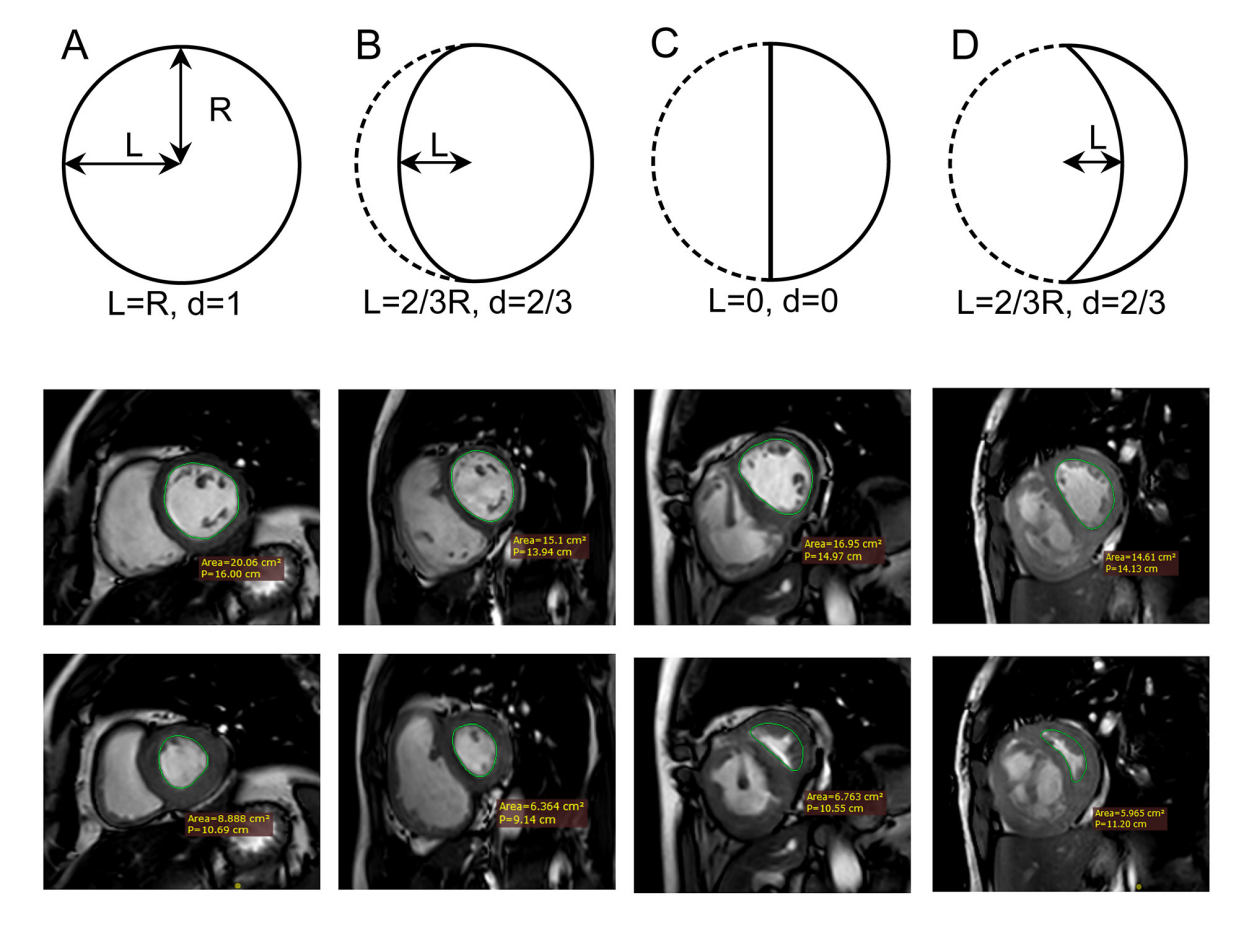
Schematic diagram and diastolic and systolic cardiac MR images of SSI calculation in patients with various mPAP values. (A) Normal mPAP; (B) mPAP of 35 mm Hg; (C) mPAP of 56 mm Hg; and (D) mPAP of 81 mm Hg. mPAP: mean pulmonary artery pressure; SSI: septum swing index. Set the distance between the midpoint of the elastic rope and the center of the circle as L, R indicates the radius; d = L/R.

We approximated the interventricular septum movement as the swinging movement of an elastic rope connected to both ends of a semicircle with a fixed radius of R (Figure 1). The elastic rope (solid line in the figure) corresponds to the area of the LV with a semicircle formation area. During ventricular septum swinging, the pressure distribution from the left and right chambers is basically uniform, so it can be assumed that the elastic rope (septum) will remain approximately in the shape of an arc fixed at the endpoints. In this swinging process, we find that there is an index involving LV area that is related only to the amount of septum movement and not to the initial radius R of the left chamber (solid line). Thus, $SSI=4\pi \times \frac{\text{ leftventriculararea }}{{\text{ leftventricularperimeter }}^{2}}.$The calculation process is as follows:

Set the distance between the midpoint of the elastic rope and the center of the circle as L and $d=\frac{L}{R},\theta =\pi -2\mathrm{atan}\left(\frac{1}{d}\right).$

When the elastic rope is at the left of the circle center:


$$SSI=\frac{2\pi^{2}+\pi \left(2\left(d-\frac{1}{d}\right)+\theta {\left(d+\frac{1}{d}\right)}^{2}\right)}{{\left(\pi +\theta \left(d+\frac{1}{d}\right)\right)}^{2}}\;.\text{When}\;L=R,SSI=1.$$

When the elastic rope is at the middle of the circle center:


$$SSI=\frac{2\pi^{2}}{(2+\pi {)}^{2}}\approx 0.745$$

When the elastic rope is at the right of the circle center:


$$SSI=\frac{2\pi^{2}-\pi \left(2\left(d-\frac{1}{d}\right)+\theta {\left(d+\frac{1}{d}\right)}^{2}\right)}{{\left(\pi +\theta \left(d+\frac{1}{d}\right)\right)}^{2}}.\text{ When L }=\text{ R, SSI }=0.$$

### Statistical analysis

Data were expressed as number, percentage, median with interquartile range, or mean with standard deviation. Parameters between groups were compared using the Mann-Whitney *U*-test or unpaired Student *t*-test for continuous data and the Chi-square test or Fisher’s exact test for categorical data.^[23, 24]^

Excluding collinearity and Bonferroni-type adjustments, binary univariate and logistic regression analyses were performed to evaluate SSI and conventional CMR predictors of PH. Receiver operating characteristics curve methodology was then used to assess the ability of SSI and conventional CMR parameters to detect PH. Sensitivity, specificity, positive predictive value (PPV), negative predictive value (NPV), and accuracy were calculated. Present estimates suggest a PH prevalence of 1% of the global population.^[25]^ After PH prevalence-adjusted, sensitivity, specificity, positive predictive value and negative predictive value are also calculated, respectively. Inter-observer correlations and agreement were determined using Pearson’s correlation coefficient and the intraclass correlation coefficient (single scoring, not adjusted).

*P* values < 0.05 were considered statistically significant. All analyses were conducted using R software, version 4.1.0 (Camp Pontanezen, TX, USA), GraphPad Prism 7.01 software (GraphPad Software, San Diego, CA, USA), and MedCalc software (MedCalc Software Ltd, Ostend, Belgium). R.J. performed the statistical analyses.

## Results

### Characteristics of the study population

**Figure 2:**
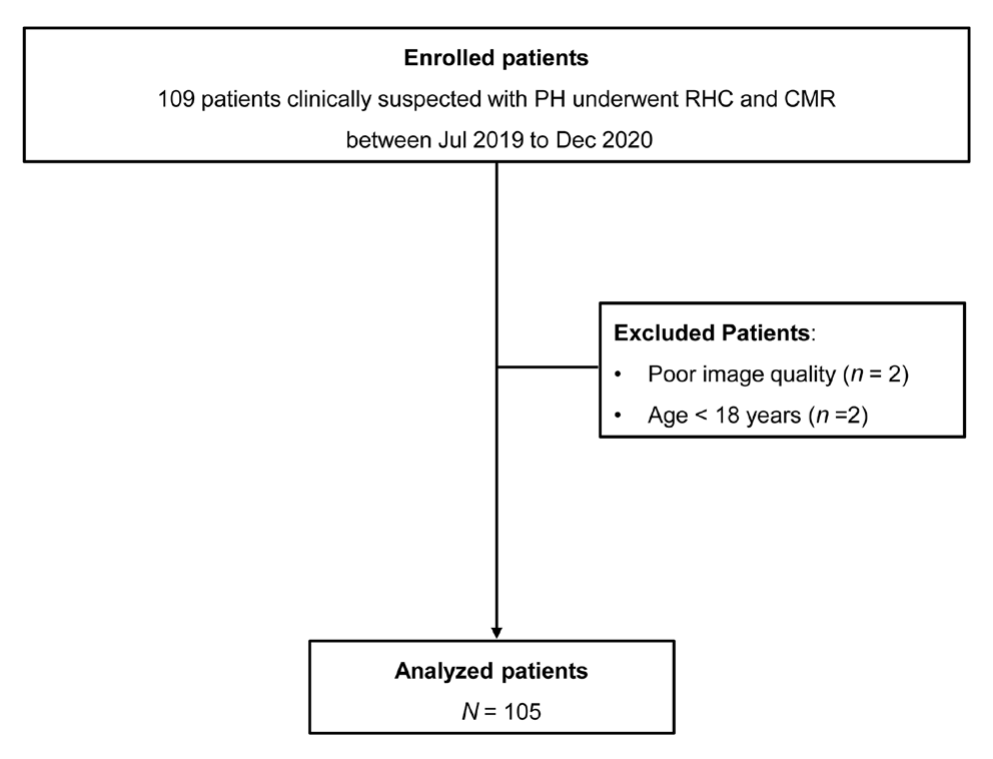
Patient flow diagram. CMR: cardiac magnetic resonance imaging; RHC: right heart catheterization.

We screened 109 consecutive patients with clinically suspected PH who underwent both RHC and CMR. A total of 105 patients were included in the final analyses (Figure 2). The included patients had a mean age of 47.8 ± 15.0 years, and 68 (64.8%) were female. Demographic information and hemodynamic measurements obtained at RHC are shown in Table 1.

**Table 1:** Baseline patient characteristics

Characteristics	mPAP < 25 mm Hg (*n* = 17)	mPAP ≥ 25 mm Hg (*n* = 88)	*P* value
Age, y	50.2 ± 13.4	45.8 ± 14.5	0.283
Female, *n* (%)	10 (58.8)	58 (65.9)	0.556
NT-proBNP, ng/L	70.9 (5.0, 1495.0)	456.8 (5.0, 5902.0)	< 0.001
6MWD, m	475.0 (275.0, 575.0)	406.0 (105.0, 645.0)	0.110
*Echocardiography*			
	39.7 ± 7.3	90.7 ± 14.2	< 0.001
TAPSE (cm)	2.4 ± 0.5	1.7 ± 0.9	0.002
ENDSEI	1.0 ± 0	1.9 ± 0.7	< 0.001
RATD (cm)	3.8 ± 0.7	4.5 ± 0.8	0.001
RALD (cm)	4.5 ± 0.6	5.3 ± 0.9	< 0.001
RVEDTD (cm)	3.4 ± 0.8	4.3 ± 0.7	< 0.001
RVEDLD (cm)	6.2 ± 0.9	6.9 ± 0.4	< 0.001
*Hemodynamics*			
mPAP (mm Hg)	18.8 ± 4.2	46.6 ± 12.7	< 0.001
PAWP (mm Hg)	7.5 ± 3.4	6.8 ± 2.7	0.358
Cardiac output (L/min)	6.7 ± 1.8	4.8 ± 2.0	< 0.001
PVR (Wood units)	1.8 ± 0.8	9.7 ± 5.5	< 0.001

Data expressed as mean ± standard deviation, median (interquartile range), or number (%). 6-MWD: 6-minute walk distance; ENDSEI: end-systolic stage eccentricity index; mPAP: mean pulmonary artery pressure; NT-proBNP: N-terminal pro-brain natriuretic peptide; PASP: pulmonary arterial systolic pressure; PAWP: pulmonary artery wedge pressure; PVR: ulmonary vascular resistance; RALD: right atrial longitudinal dimension; RATD: right atrial transverse dimension; RVEDLD: right ventricular end-diastolic longitudinal dimension; RVEDTD: right ventricular end-diastolic transverse dimension; TAPSE: tricuspid annular plane systolic excursion.

### Relationship between mPAP and quantitative CMR parameters

**Figure 3:**
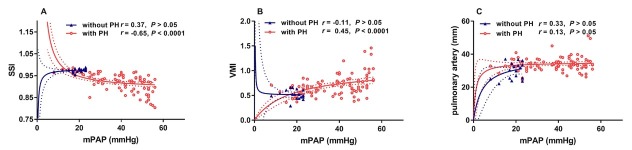
Spatially matched correlations between mPAP, SSI, VMI, and mean pulmonary artery diameter. Red circles: patients with PAH; black triangles: patients without PAH. Red and blue dotted lines represent the 95% confidence interval, respectively. mPAP: mean pulmonary arterial pressure; PH: pulmonary hypertension; *r*: Spearman rank correlation coefficient; SSI: septum swing index; VMI: ventricular mass index.

Conventional CMR-derived morphologic and functional parameters related to PH are shown in Table 2. Structure and function parameters of both the LV and RV differed significantly between patients with and without PH. Conventional parameters for detecting PH, such as main pulmonary artery diameter and VMI were significantly higher, whereas SSI was significantly lower in patients with PH. SSI and mPAP were negatively correlated in the whole study population and in patients with PH but not in patients without PH (Figure 3A). By contrast, VMI and mPAP were positively correlated in the whole study population and in patients with PH (Figure 3B). While main pulmonary artery diameter and mPAP were positively correlated in the whole study population (Spearman rank correlation coefficient = 0.307, *P* = 0.001), they were not significantly correlated in patients with PH or without PH (Figure 3C). Both inter-observer and intra-observer agreement were excellent for SSI (intraclass correlation coefficient = 0.970 and 0.930, respectively, both *P* < 0.001).

**Table 2:** Cardiac magnetic resonance imaging parameters in patients with or without pulmonary arterial hypertension

Parameter	mPAP < 25 mm Hg (*n* = 17)	mPAP ≥ 25 mm Hg (*n* = 88)	*P* value
LV ejection fraction (%)	59.3 ± 7.9	53.5 ± 11.4	0.031
End-diastolic volume (mL)	150.2 ± 29.3	110.7 ± 30.7	< 0.001
End-systolic volume (mL)	61.9 ± 19.4	51.3± 18.8	0.031
LV end-diastolic volume index (mL/m^2^)	90.1 ± 16.8	67.6 ± 17.7	< 0.001
LV end-systolic volume index (mL/m^2^)	37.2 ± 12.1	31.1 ± 10.4	0.040
LV end-diastolic mass index (g/m^2^)	50.2 ± 7.2	47.5 ± 9.0	0.133
LV end-systolic mass index (g/m^2^)	51.9 ± 6.7	46.2 ± 9.2	0.003
Main pulmonary artery diameter (mm)	29.9 ± 5.0	34.0 ± 4.3	0.005
RV stoke volume (mL)	63.4 ± 18.1	47.4 ± 24.9	0.002
RV ejection fraction (%)	42.6 ± 9.7	26.1 ± 13.1	< 0.001
End-diastolic volume (mL)	150.0 ± 31.7	195.0 ± 71.1	0.011
End-systolic volume (mL)	86.7 ± 25.3	147.6 ± 66.3	< 0.001
RV end-diastolic volume index (mL/m^2^)	89.9 ± 18.3	118.1 ± 38.5	0.003
RV end-systolic volume index (mL/m^2^)	52.3 ± 15.9	88.9 ± 36.0	< 0.001
RV end-diastolic mass index (g/m^2^)	25.6 ± 4.6	32.7 ± 8.7	< 0.001
RV end-systolic mass index (g/m^2^)	19.5 ± 4.5	31.3 ± 10.6	< 0.001
VMI	0.52 (0.46, 0.61)	0.69 (0.57, 0.80)	< 0.001
SSI	0.97 (0.97, 0.98)	0.93 (0.90, 0.96)	< 0.001

Data expressed as mean ± standard deviation or median (interquartile range). LV: left ventricle; RV: right ventricle; SSI: septum swing index; VMI: ventricular mass index.

### Factors predicting PH

Table 3 shows the results of univariate binary logistic regression analysis, including unadjusted odds ratios for PH for each variable. After adjusting by Bonferroni P values, PH remained significantly associated with main pulmonary artery diameter, LV end-diastolic volume index, RV ejection fraction, RV end-systolic mass, RV end-systolic volume index, RV end-systolic mass index, and SSI. After assessing collinearity of variables in linear regression analysis of the association between CMR with mPAP, we excluded LV end-diastolic volume index and RV end-systolic mass index. The remaining factors (main pulmonary artery diameter, RV ejection fraction, RV end-systolic mass, RV end-systolic volume index, and SSI) that were significant after Bonferroni adjustment were then entered into stepwise multivariable binary logistic regression to analyze predictors of PH. This revealed SSI as an independent predictor of PH (adjusted odds ratio: 12.9, 95% confidence interval: 3.6 to 45.5, *P* = 0.003).

**Table 3:** Univariable analyses of cardiac magnetic resonance imaging predictors of pulmonary arterial hypertension

Parameter	Odds ratio (95% CI)	*P* value	Bonferroni *P* value
Main pulmonary artery diameter	1.27 (1.10, 1.46)	0.001	0.018
LV ejection fraction	0.94 (0.89, 0.99)	0.044	0.792
LV end-diastolic mass	0.98 (0.95, 1.01)	0.21	> 0.99
LV end-systolic mass	0.97 (0.94, 0.99)	0.033	0.594
LV end-diastolic volume index	0.94 (0.91, 0.97)	< 0.001	< 0.001
LV end-systolic volume index	0.95 (0.91, 0.99)	0.041	0.738
LV end-diastolic mass index	0.97 (0.91, 1.02)	0.245	> 0.99
LV end-systolic mass index	0.94 (0.89, 0.99)	0.025	0.45
RV stoke volume	0.98 (0.96, 1.0)	0.019	0.342
RV ejection fraction	0.89 (0.85, 0.96)	< 0.001	< 0.001
RV end-diastolic mass	1.08 (1.02, 1.14)	0.007	0.126
RV end-systolic mass	1.11 (1.04, 1.18)	0.001	0.018
RV end-diastolic volume index	1.03 (1.01, 1.06)	0.006	0.108
RV end-systolic volume index	1.05 (1.02, 1.09)	0.001	0.018
RV end-diastolic mass index	1.08 (1.02, 1.14)	0.007	0.126
RV end-systolic mass index	1.11 (1.04, 1.17)	0.001	0.018
VMI^†^	5.14 (1.65, 16.0)	0.005	0.090
SSI^‡^	11.964 (3.44, 41.61)	< 0.001	< 0.001

^†^Continuous variables were transformed into binary variables stratified by 0.6. ^‡^Variables were categorized according to interquartile ranges (25^th^ to 75^th^). LV: left ventricle; RV: right ventricle; SSI: septum swing index; VMI: ventricular mass index.

**Figure 4:**
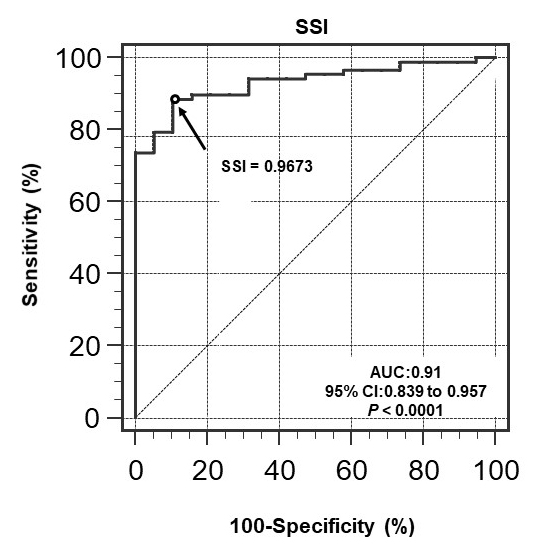
Receiver operating characteristics curve showing that SSI has good diagnostic accuracy for predicting pulmonary hypertension (mPAP ≥ 25 mm Hg). AUC: area under the receiver operating characteristics curve; mPAP: mean pulmonary arterial pressure; SSI: septum swing index.

The area under the receiver operating characteristics curve for SSI as a method of detecting PH was 0.91 (Figure 4). A cut-off SSI value of 0.9673 yielded the best balance of sensitivity (86.4% [76/88]), specificity (88.2% [15/17]), PPV (97.4 % [76/78]), NPV (55.6% [15/27]), and accuracy (86.7% [91/105]) for detecting mPAP ≥25 mm Hg. After PH prevalence-adjusted, sensitivity, specificity, PPV and NPV are 86.4%, 88.2%, 6.9%, and 99.8%, respectively.

## Discussion

This study showed that the septum swing index determined by cardiac MRI provides a simple quantitative method that links the degree of ventricular chamber deformation and mean pulmonary arterial pressure and accurately detect PH.

By numerical simulation, we can obtain a correlation elastic curve between the amplitude of an elastic rope moving from side to side and SSI. The horizontal coordinates are the ratio of the swing amplitude of the middle point of the elastic rope (relative to the initial position) to the radius of the initial circumference, and the ordinate is SSI. This curve shows that SSI changes synchronously from 1 to 0 as the septum swings from side to side. Theoretically, there is a strong correlation between the swing amplitude and PAP, so we predict that SSI and PAP will also be correlated, and when PAP is higher, SSI changes will be more obvious.

SSI has several advantages for clinical use. First, it has good sensitivity and specificity. CMR imaging constitutes one of the most complete diagnostic modalities for diagnosing PH, as it evaluates both morphology and hemodynamics of the pulmonary artery and RV. Several cine steady-state free-precession–derived parameters (RV end-diastolic volume index or RV stroke volume index) and phase-contrast regional area changes have been suggested as powerful biomarkers for use in prognosis and treatment. RV end-diastolic volume index ≥ 84 mL/m^2^, RV end-systolic volume index ≥ 70 mL/m^2^, RV stroke volume index < 25 mL/m^2^, LV end-diastolic volume index < 40 mL/m^2^, and RV mass index > 59 g/m^2^ have all been associated with worse prognosis. Previous studies have reported that pulmonary flow artifact can be used to predict PH, with a sensitivity of 86%, specificity of 85%, and positive predictive value of 95%. Other reports found that VMI had a sensitivity of 81%–98% and specificity of 69%–89%, with an optimal cut-off value of 0.45,^[26]^ and pulmonary artery mean velocity > 11.7 cm/sec had a sensitivity of 92.9% and specificity of 82.4%.^[27]^ By comparison, SSI has a high sensitivity (86.4%), specificity (88.2%), and positive predictive value (97.4%) for detecting PH, with an optimal cut-off value of 0.9673.

**Table 4:** Comparisons of the area under the curve of cardiac magnetic imaging for detecting pulmonary hypertension

	AUC	SE	95%CI	*P* value
SSI	0.919	0.0275	0.849–0.963	< 0.0001
main pulmonary artery diameter	0.715	0.0777	0.618–0.800	0.006
RV end-systolic volume index	0.832	0.0439	0.746–0.898	< 0.0001
VMI^†^	0.513	0.0652	0.413–0.612	0.840

^†^Continuous variables were transformed into binary variables stratified by 0.6. SSI: septum swing index; VMI: ventricular mass index; AUC: area under the receiver operating characteristics curve; SE: standard error: CI: Confident interval.

Secondly, calculating SSI is a simple procedure. SSI values are determined by measuring the maximum or minimum area and diameter during diastole and systole on short-axis cine images and then inserting these measurements into a fixed formula. Measuring diastolic and systolic area and diameter is the most basic CMR technique, with low technical difficulty. Conversely, CMR image processing techniques based on steady state free preceesion (SSFP) sequences generally require independent and complex software.

Another advantage of SSI is that it reflects cardiac remodeling, combining right and left heart interactions. The right heart affects left heart function through the interventricular septum. Finally, SSI has broad clinical potential for predicting prognosis and assessing the severity of right heart dysfunction.^[28]^ It may be combined with PH risk grades for prognosis prediction and may be used to explore correlations with other pulmonary vascular remodeling parameters, such as pulmonary vascular resistance, cardiac output, and mixed venous oxygen saturation.

Based on its numerical approximation, SSI is a function related to only L/R, representing the swing distance divided by the LV radius. The rationale for not measuring these two lengths directly is because of measuring reproducibility. When measuring a distance, two exact points must be chosen. These points are difficult to define or reproduce when the target region is the chamber of a cardiac ventricle, which is not uniform in shape and contains chordae tendineae. By contrast, drawing a smooth contour inscribing the LV chamber is intuitively easy to accomplish. Our results showed that SSI values were determined consistently between reviewers.

Although averaged over two slices, SSI determination is based mostly on 2-dimensional information, while the actual septum moves in three dimensions. In the future, we plan to conduct studies including long-axis cine images to cover more LV mass or studies using a multi-slice weighted average algorithm, which may correlate even more strongly with mPAP. To reduce the radiologist’s workload and increase data repeatability, automatic LV chamber fitting and an automated algorithm for SSI calculation may also be developed.

Our numerical approximation also showed that SSI is positively correlated with the L/R ratio but not in a linear manner. When the septum swung from the left to the middle, SSI only changed from 1 to 0.745, but when the septum swung from the middle to the right, it decreased more rapidly to 0. This phenomenon suggests that in addition to discriminating patients with PH from those without PH, SSI may be especially sensitive for detecting changes in patients with severe PH during treatment or long-term follow-up.

In the study, SSI was proved to be with satisfying AUC, sensitivity, specificity, PPV, NPV and accuracy for detecting PH. After PH prevalence –adjusted, sensitivity, specificity and AUC still remained. However, PPV had descended from 97.4% to 6.9%, and NPV had increased from 55.6% to 99.8%. Because PH is a rare disease, the prevalence rate is only 1%. The low prevalence rate has a significant impact on both PPV and NPV. For the general population, SSI has a high NPV, but a low PPV. However, at the hospital level, patients have symptoms to seek medical advice, so the probability of PH will also be greatly increased, so it is not suitable for screening of natural populations. For patients with suspected PH symptoms, SSI is still of great diagnostic value, because the sensitivity and specificity are relatively high, close to 90%.

In conclusion, septum swing index-derived from CMR was lower in patients with PH and is a simple, reliable method for predicting PH.

## Limitations

First, our study was a retrospective cross-sectional study, and the negative control group did not have as many samples. The CMR-derived SSI is newly invented by us. This was an exploratory study, and we plan to carry out a prospective large sample study to further study it. Secondly, different degrees of severity should elicit different patterns of RV response. In fact, in cases of mild PH short-termed it should be less likely that a significant RV pressure overload is present leading to RV hypertrophy or geometrical changes in the contraction pattern. Thirdly, the SSI has good diagnostic accuracy for predicting PH, the actual SSI values between “PH” and “no PH” are extremely close, with not good enough sensitivity.
